# Investigation of Shear-Driven and Pressure-Driven Liquid Crystal Flow at Microscale: A Quantitative Approach for the Flow Measurement

**DOI:** 10.3390/mi12010028

**Published:** 2020-12-29

**Authors:** Jianqin Zhu, Runze Tang, Yu Chen, Shuai Yin, Yi Huang, Teckneng Wong

**Affiliations:** 1 National Key Laboratory of Science and Technology on Aero-Thermodynamics, School of Energy and Power Engineering, Beihang University, Beijing 100083, China; zhujianqin@buaa.edu.cn (J.Z.); buaatrz@163.com (R.T.); chenyuss@buaa.edu.cn (Y.C.); 2 School of Mechanical and Aerospace Engineering, Nanyang Technological University, 50 Nanyang, Singapore 639798, Singapore; YI0001AI@e.ntu.edu.sg; 3 Research Institute of Aero-Engine, Beihang University, Beijing 100083, China

**Keywords:** liquid crystal, shear flow, director field, flow measurement, flow visualization

## Abstract

The liquid crystal-based method is a new technology developed for flow visualizations and measurements at microscale with great potentials. It is the priority to study the flow characteristics before implementation of such a technology. A numerical analysis has been applied to solve the simplified dimensionless two-dimensional Leslie–Ericksen liquid crystal dynamic equation. This allows us to analyze the coupling effect of the LC’s director orientation and flow field. We will be discussing two classic shear flow cases at microscale, namely Couette and Poiseuille flow. In both cases, the plate drag speed in the state of Couette flow are varied as well as the pressure gradients in Poiseuille flow state are changed to study their effects on the flow field distributions. In Poiseuille flow, with the increase of applied pressure gradient, the influence of backflow significantly affects the flow field. Results show that the proposed method has great advantages on measurement near the wall boundaries which could complement to the current adopted flow measurement technique. The mathematical model proposed in this article could be of great potentials in the development of the quantitatively flow measurement technology.

## 1. Introduction

Thermotropic liquid crystals (LCs) are a mesophase exhibiting the appearance of an anisotropic liquid whose molecules represent in rodlike or discotic shape [1]. Its unique features are the temperature dependent nature, whose phase transitions are controlled by the temperature. For thermotropic LCs, the liquid crystals in nematic phase attracts tremendous attention [2]. Among them, the rod like molecules with large length-to-diameter ratio, and no long range order in the molecular centroid are the most common ones. Due to the presence of the rod-shaped molecules, the fluid translational motion is coupled with the internal orientational motion of molecules. Hence, their flow properties can be much richer than simple Newtonian fluids. Conversely, the change of molecular orientation order affects the flow in return. This coupling has important practical consequences upon the application of LCs [3]. Therefore, investigations on mechanism of hydrodynamic shear induced reorientation of the liquid crystal molecules are necessary. The research of the T.G. Anderson team focuses on the physical change of liquid crystal fluid under microfluid channel with pressure gradient [4]. The team studies the transformation process of the two states of liquid crystal fluid by numerical calculation and compares the results of the transition of the flow state with the experiment of the Sengupta team [5]. The dimensionless equation is obtained through normalization, which makes the results more intuitive and more universal. In addition, the elastic free energy of liquid crystals in different states is presented. The reasons for the change of liquid crystal flow state are well explained by the comparison of the elastic free energy [5]. Compared to the fluid flow at marcoscale, flow at microscale (mostly in microchannels here) is fundamentally different due to the limited inertial effects and dominating viscous stress and interfacial tension.

In this paper, we choose 4-Cyano-4′-pentylbiphenyl (5CB) as which stays in nematic phase under room temperature. 5CB is a type of nematic liquid crystal (NLC) called “calamitics” [6] which represents the rod-like molecular structure. The structure can be observed experimentally using the polarized microscopy configuration. Recent literatures have been focusing more on the characteristic response to the electric field [7,8,9,10]. The well-known orientation Fredericks transition has been extensively studied and applied in practical applications [11]. Furthermore, the combination of the delicate microscale fluidic control with LCs, especially the nematic LCs has allowed the possibility of topological studies [12], phase transitions [13,14,15,16]and the unique stripe structure [17,18]. More importantly, it also leads to the diverse application in fields of microfabrications, 3D printing and bioengineering [12,13,19,20]. Nonetheless, very few works have been reported on the reorientation influenced by the hydrodynamic pressure at microscale [21,22,23]. Currently, the most commonly used model to study directional vector deflection and flow of nematic liquid crystals is the Leslie–Ericksen equations (L-E equation). We apply the Leslie–Ericksen formalism for nematodynamics to investigate the shear flow model. First, we describe the model of the liquid crystal by means of L-E equations [24]. The analysis is restricted to the steady state deformations of the nematic phase where α_3_/α_2_ > 0. The flow behavior of nematic liquid crystals can be generally categorized into two major types through the signs of the two Leslie viscosities α_2_ and α_3_. If α_3_/α_2_ > 0, then the liquid crystal is flow aligning, otherwise it is flow tumbling [25]. Next, we addressed two typical steady 2D cases: the shear-driven (Couette) and the pressure-driven (Poiseuille) flow of nematic liquid crystal in a parallel microchannel respectively. Then we applied justified assumptions for simplification purpose before solving them numerically. The director profile is calculated for various pressure gradients and shear stresses. For pressure-driven flow cases, we find that under the limitation of strong anchoring and weak flow effects, flow alignment is not presented. In fact, the director field is majorly determined by the boundary conditions. For other cases, the results clearly show the influence of flow condition on reorientation of director field, which provide guideline for flow measurements at microscale [26,27,28].

## 2. Mathematical Models

There are two major differences considering the hydrodynamics of simple Newtonian fluids and that of the LCs. First, LCs’ molecules can be rotated by the pressure gradient due to its unique geometries. Second, the equilibrium free energy of LCs is more complex. This coupling between the elastic energy and the flow, which is called backflow, leads to rich hydrodynamic behaviors. In order to seize the main physical properties of LCs, the details of the molecules are neglected and the ideal approximation is processed. For example, for nematic LCs, LC molecules are often idealized as a long rod with a symmetric head and tail, and hence uniaxial symmetry. As mentioned earlier, LCs molecules are anisotropic, therefore we need to introduce variables called order parameter to describe the alignment and orientation of LCs. We adopt the well accepted Leslie–Ericksen model to describe the director orientation and LC flow. In the continuum theory, we define the orientation of the LCs at a point by a unit vector **n** called the director.

The full equation of nematodynamics consists of

(1) $$V_{i,i}=0$$

(2) $$\rho {\vec{v}}_{i}=\rho F_{i}-{\left(p+W_{F}\right)}_{,i}+{\tilde{g}}_{j}n_{j,i}+G_{j}n_{j,i}+{\tilde{t}}_{ij,j}$$

(3) $${\left(\frac{\partial W_{F}}{\partial n_{i,j}}\right)}_{j}-\frac{\partial W_{F}}{\partial n_{i}}+{\tilde{g}}_{i}+G_{i}=\lambda n_{i}$$

**Figure 1:**
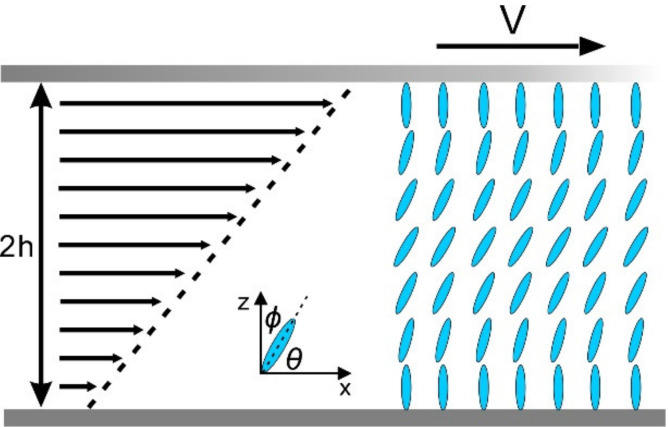
Schematics and description of the mathematical model of a nematic liquid crystal in the Couette flow. The upper plate is moved to the right at a constant velocity.

**Figure 2:**
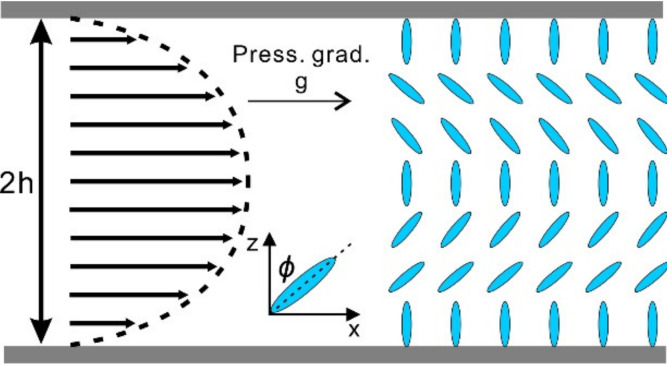
Schematics and the mathematical description of a nematic liquid crystal in the Poiseuille flow.

Representing mass, energy, and momentum conservations respectively. We consider a steady 2D plane Poiseuille–Couette flow of nematic liquid crystal in a microfluidic channel with two parallel boundaries and a thin film geometry. The upper plate travels along the x direction at a constant speed *V* (shear-driven flow) or a constant pressure gradient is applied within the LCs (pressure-driven flow) as shown in Figure 1 and Figure 2 respectively.

### 2.1. Couette Flow

It will be assumed that the director and the velocity take the forms

(4) $$\vec{n}=[\cos\theta (z),0,\sin\theta (z)]$$

(5) $$\vec{v}=[v(z),0,0]$$

We now investigate the coquette flow at a fixed distance, *d* = 2h where the lower plate is at rest and the upper plate is moving at a constant velocity V as shown in Figure 1. Three assumptions are listed as follows (Macsithigh, G. P, and P. K. Currie, 1977):

The one-constant approximation applied to the Oseen–Zocher–Frank elastic free energy equation. In details *K_11_* = *K_22_* = *K_33_* ≡ *K*, where *K_11_* is the splay coefficient, *K_22_* is the twist coefficient, and K_33_ is the bend coefficient. (Currie, P. K., and G. P. Macsithigh, 1979).

Parodi’s relation [29]

(6) $$\alpha_{6}=\alpha_{5}+\alpha_{3}+\alpha_{2}$$

The zero viscosity coefficient${\;\alpha }_{1}=0$.

From the (2), (3) and the assumptions, it follows that

(7) $$\frac{dv}{dz}=\frac{c}{g(\theta )}$$

(8) $$2K\frac{d^{2}\theta }{dz^{2}}-c\frac{\left[\gamma_{1}+\gamma_{2}\cos(2\theta )\right]}{g(\theta )}=0$$

(9) $$g(\theta )=\frac{1}{2}[\alpha_{5}-\alpha_{2}+\alpha_{4}+2(\alpha_{3}+\alpha_{2})\cos^{2}\theta ]$$

For simplicity we nondimensionalize the equations by using the scaling lengths with half-width of the channel *h*, the velocity $\bar{v}$ with $\bar{v}=V/2$, and all viscosities $\gamma_{i}\;\alpha_{i}$ with$\;\alpha_{4}$. The dimensionless governing equations become:

(10) $$\frac{d^{2}\theta }{dz^{2}}=\frac{Vh\alpha_{4}}{4K}\frac{\left(\alpha_{5}+1+\alpha_{2}+2\alpha_{3})(\gamma_{1}+\gamma_{2}\cos2\theta \right)}{\alpha_{5}+1-\alpha_{2}+2(\alpha_{2}+\alpha_{3})\cos^{2}\theta }$$

(11) $$\frac{d^{2}v}{dz^{2}}=\frac{(\alpha_{5}+1+\alpha_{2}+2\alpha_{3})\sin2\theta }{{\left[\alpha_{5}+1-\alpha_{2}+2(\alpha_{2}+\alpha_{3})\cos^{2}\theta \right]}^{2}}\theta^{\prime }$$

where $\gamma_{1}=\alpha_{3}-\alpha_{2\;,\;}\gamma_{2}=\alpha_{6}-\alpha_{5}$, the $\alpha_{i}$ are constant viscosities for the NLC. The director is strongly anchored parallel to the plates at the boundaries (Leslie, F.M. 1979), and that the solution for *Φ* is symmetric in *z*. A more detailed derivation process can be refereed to Appendix A and Appendix B for Couette flow.

We applied the boundary conditions as: $v\left(-1\right)=0$, $v\left(1\right)=2$,$\;\theta \left(-1\right)=\theta \left(1\right)=П$/2.

### 2.2. Poiseuille Flow

Here we consider the steady 2D Poiseuille flow in a microfluidic channel −h≤z≤h as schemed in Figure 2. To investigate a typical, two-dimensional shear flow behavior, the director and the velocity take the forms

(12) $$\vec{n}=[\sin\phi (z),0,\cos\phi (z)]$$

(13) $$\vec{v}=[v(z),0,0]$$

In order to simplify the boundary conditions, it is assumed that *Φ* is the angle between z axis and the molecule axis (Figure 2). We use the first two assumption $\alpha_{6}=\alpha_{5}+\alpha_{3}+\alpha_{2}$ and K1=K3=K. Therefore, the following formulas can be obtained

(14) $$\frac{dv}{dz}=\frac{c}{g(\phi )}$$

(15) $$2K\frac{d^{2}\phi }{dz^{2}}+c\frac{\left[\gamma_{1}-\gamma_{2}\cos(2\phi )\right]}{g(\phi )}=0$$

(16) $$g(\phi )=\frac{1}{2}[2\alpha_{1}\sin^{2}\phi \cos^{2}\phi +(\alpha_{5}-\alpha_{2})\cos^{2}\phi +(\alpha_{3}+\alpha_{6})\sin^{2}\phi +\alpha_{4}]$$

where $\gamma_{1}=\alpha_{3}-\alpha_{2\;,\;}\gamma_{2}=\alpha_{6}-\alpha_{5}$, the $\alpha_{i}$ are constant viscosities for the NLC.

To nondimensionalize the equations, we adopted the following strategies: scale the physical length with the half width of the channel *h*, the velocity *v* with $\bar{v}=2Gh^{2}/3\alpha_{4}\;$(Anderson, T. G. et al., 2015), and all viscosities $\gamma_{i},\;\alpha_{i}$ with$\;\alpha_{4}$. The dimensionless form of the governing equations become,

(17) $$\frac{\mathrm{dv}}{dz}=-\frac{3z}{\left[2\alpha_{1}\sin^{2}\phi \cos^{2}\phi +\left(\alpha_{5}-\alpha_{2}\right)\cos^{2}\phi +\left(\alpha_{3}+\alpha_{6}\right)\sin^{2}\phi +1\right]}$$

(18) $$\frac{\phi ’’\left(z\right)}{\gamma_{1}-\gamma_{2}\cos2\phi }\left[2\alpha_{1}\sin^{2}\phi \cos^{2}\phi +\left(\alpha_{5}-\alpha_{2}\right)\cos^{2}\phi +\left(\alpha_{3}+\alpha_{6}\right)\sin^{2}\phi +1\right]=gz$$

where *g* = Gh^3^/K is a dimensionless pressure gradient.

It is assumed that the director is strongly anchored vertically and uniform to the plates at the boundaries before the flow turned on and the solution for Φ is symmetric in z.

**Figure 3:**
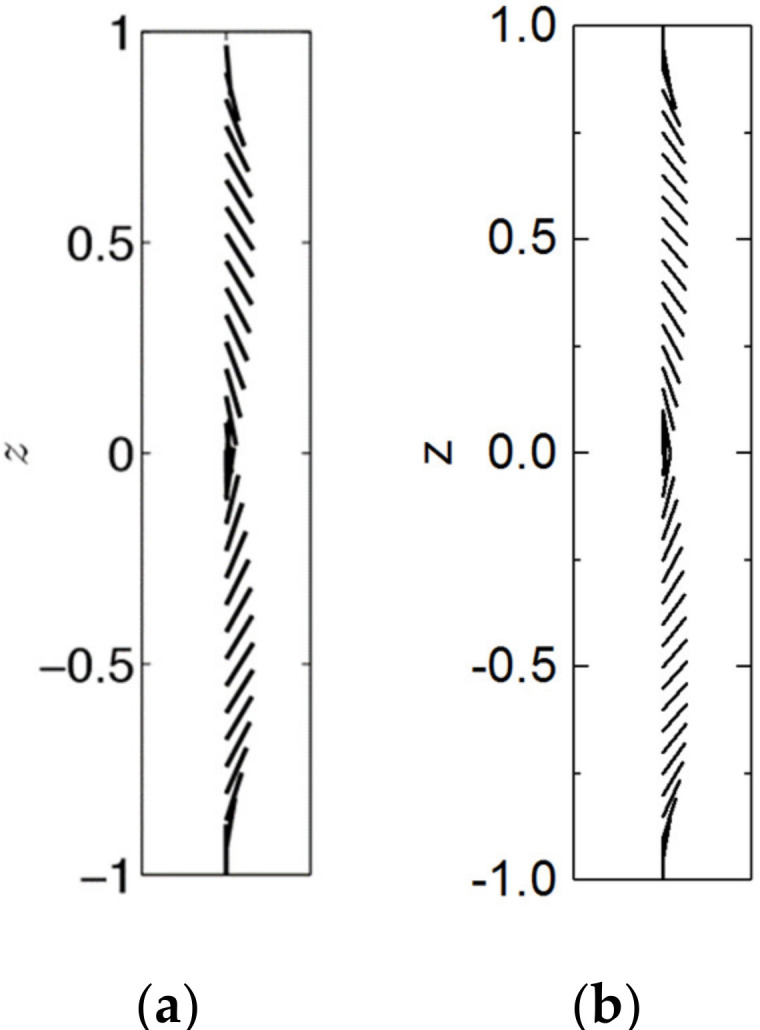
(**a**) Weak flow director profile across the dimensionless channel width −1≤ z ≤ 1 under strong anchoring and *g* = 25 (Anderson, T. G. et al., 2015). (**b**) The calculated director profile of strong anchoring at *g* = 25.

The director profile across the dimensionless channel width −1≤ *z* ≤ 1 under strong anchoring and *g* = 25 is calculated in Figure 3a to compare with the results obtained by Anderson team (Anderson, T. G. et al., 2015) in Figure 3b. The calculated deflection angle of liquid crystal is in good agreement with the results in the research content of Anderson team.

## 3. Results

We compute solutions of the system by choosing parameters for the liquid crystal 5CB (Sengupta, A, et al., 2013) in the nematic phase. The dimensionless viscosities chosen are $\alpha_{1}=-0.1594$, $\alpha_{2}=-0.9859$, $\alpha_{3}=-0.0535$, $\alpha_{5}=0.7324$, $\alpha_{6}=-0.3944$, $K=4\times 10^{-12}\;N$; and $\gamma_{1}=\alpha_{3}-\alpha_{2}$, $\gamma_{2}=\alpha_{6}-\alpha_{5}$. Equation is solved using fourth-order Runge–Kutta integration and the shooting method.

### 3.1. Couette Flow

In this section, we conducted a systematic parametric investigation on velocity and director fields of a nematic liquid crystal under the shear controlled by the velocity *V* of the upper plate. The height of the channel was setup as 10 µm. The computations were carried out for different velocities of the upper plane.

**Figure 4:**
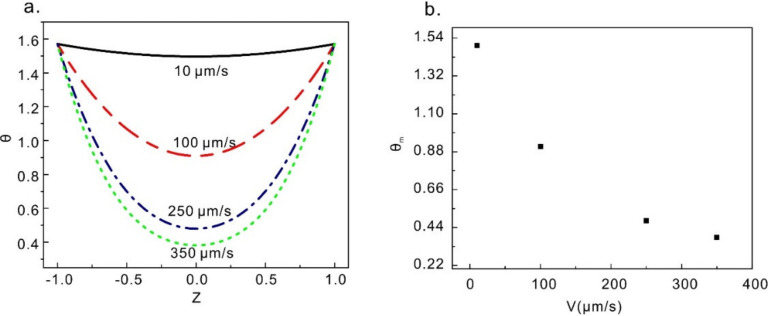
(**a**) Director profiles at different velocities of the upper plate. (**b**) The maximum directional angle at different velocities of the upper plate.

Figure 4a shows director profiles when the velocity of the upper plate is 10 µm/s, 100 µm/s, 250 µm/s, and 350 µm/s respectively. It can be seen in Figure 4a that the director profile is almost an axisymmetric parabola at the center. With increasing of the velocity, the angle of the director becomes smaller which is more flow orientated. In Figure 4b, the mid-plane angles $\emptyset_{m}\;$are plotted as functions of velocity *V*. We found that the mid-plane angles $\emptyset_{m}$ tend to $\emptyset_{c}$=arctan[(α_3_/α_2_)^0.5^] ($\emptyset_{c}$= 0.22, in this article) with the increasing velocity, which agrees with the continuum theory proposed by Leslie and Ericksen [30]. Leslie defined the Leslie angle as: “for which there is no hydrodynamic torque on the director in simple shear flow of an infinitely thick sample”. It is found in this article that the Leslie angle still exists in microchannel.

### 3.2. Poiseuille Flow

**Figure 5:**
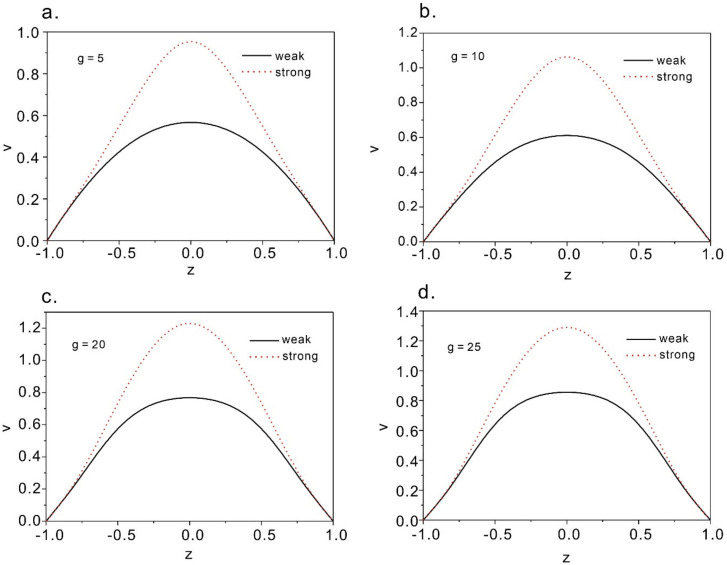
Strong and weak flow solutions at different dimensional pressure gradients. (**a**–**d**) correspond to the condition of g = 5, 10, 20, and 25 respectively.

Figure 5 shows the solutions at Poiseuille flow with the change of dimensionless pressure gradient *g*. It can be found that for both weak flow and strong flow anchoring boundary conditions the solutions exist for all flow rates (Figure 5). From T.G. Anderson’s founding on the elastic energies of the two solutions, there is a critical dimensional pressure gradient g*. The energy is lower when the value of *g* is lower. Therefore, it explains that for cases of g < g*, the weak flow would occur, while for the cases of g > g* the strong flow solution would be expected. In this part, g* is around 40 for strong anchoring boundary condition.

**Figure 6:**
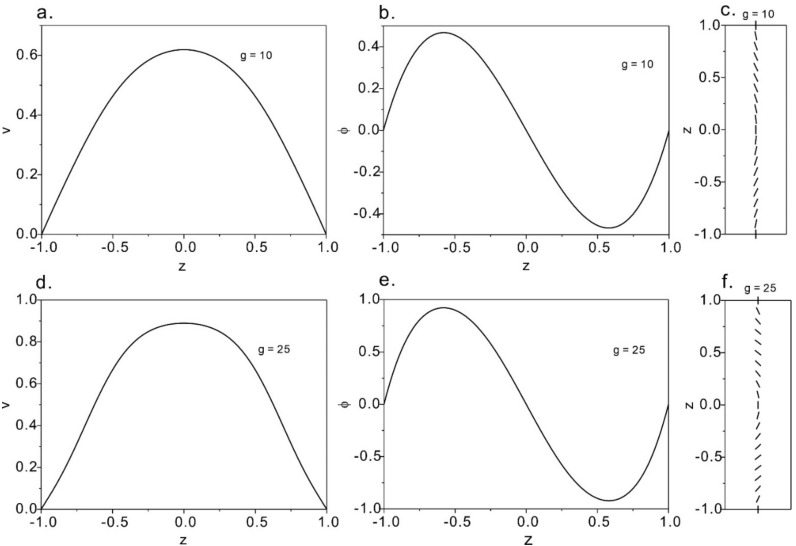
Velocity profiles and director fields at strong anchoring condition. (**a**–**c**): The solution of strong anchoring at g = 10. (**a**) Velocity distributions over the channel width; (**b**) angle of the director; (**c**) director distributions over the microchannel. (**d**–**f**): Director and flow distributions within the microchannel under the pressure of *g* = 25.

Figure 6 shows weak flow solution at *g* = 10 and *g* = 25. We find out that the solution to the anchoring-dominated case has two regions symmetrically placed around the channel centerline. Near the walls and at the centerline, the director follows the strong homeotropic anchoring condition. The velocity profiles are approximately parabolic. The greater the dimensionless pressure gradient is, the greater the velocity and the perturbation of the director are.

**Figure 7:**
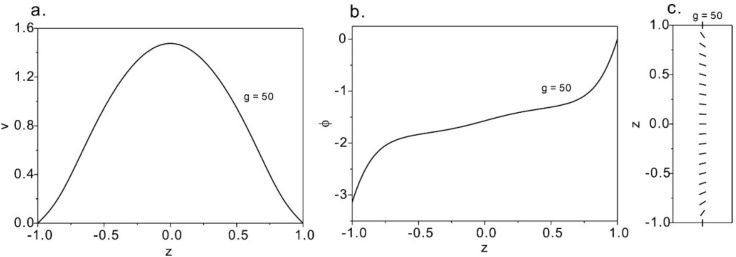
The solution of strong solution at *g* = 50. (**a**) The velocity profile; (**b**) angle of the director; (**c**) the distribution of the director at *g* = 50.

Strong flow solutions at *g* = 50 are plotted in Figure 7, where we see more complex phenomena. The velocity front is a sharper parabolic shape and the director profile is also symmetrical. This differs from the weak flow, where the direction filed is mostly aligned with the flow, regulated by the homeotropic anchoring only near the walls. Therefore, when the pressure gradient further increases, the nematic profile evolves into a flow-aligned state where the director field is majorly flow-aligned. For weak flow, the director orientates normally to the flow orientation at the center w while for strong flow, the director aligns along the flow direction at the center region.

Above, we discussed two kinds of boundary conditions. However, during numerical investigations, a third occurrence may happen, arising from the boundary condition$\;\Phi \left(-1\right)=\pi$ instead of the condition$\;\Phi \left(-1\right)=-\pi$. The boundary conditions drive the director to rotate through an angle + π across the channel, but the flow near the wall pushes the director in the opposite direction.

## 4. Discussion

**Figure 8:**
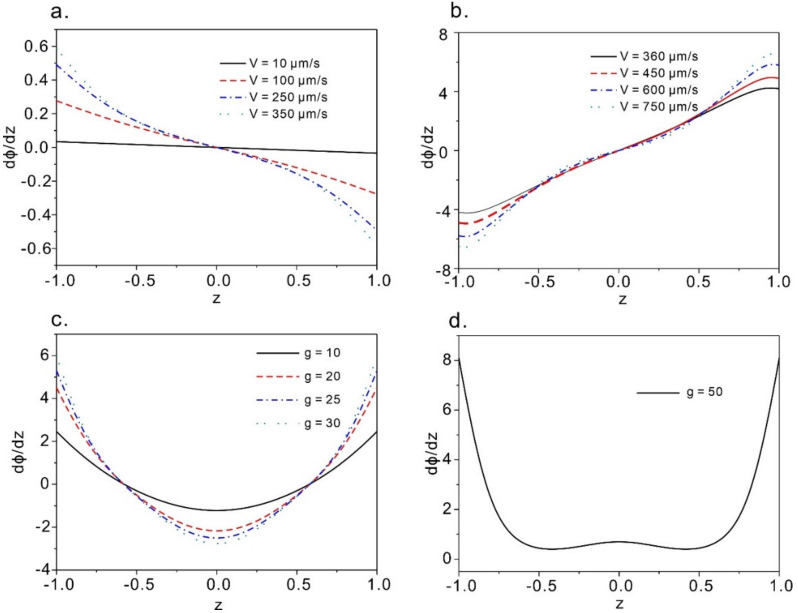
Positional sensitivity of the directional profile of the LC flow. (**a**) and (**b**) are sensitivities of the LC in Couette flow at various velocities of the upper plate; (**c**) and (**d**) represent the sensitivities at various dimensional pressure gradient in Poiseuille flow.

We have calculated the derivative of the directional profile with respect to the channel positions, which represents the sensitivity of the proposed method for flow measurement, as shown in Figure 8. The results indicate that for both the Couette flow and Poiseuille flow, the maximum sensitivity always occurs near the wall boundary (that is the two plates), where the traditionally adopted tracing particle-based measurement technique (namely particle image velocimetry) shows vulnerability. In the cases of Couette flow, we can always find the highest sensitivities near the two plates (see Figure 8a,b) and the lowest sensitivity in the middle region. Similarly, we obtained the maximum sensitivities near the two boundaries (Figure 8c,d). However, when it is under the relatively weak pressure gradient for Poiseuille flow (g = 10~30), Figure 8c indicates a good sensitivity can be obtained in the middle region while for a strong flow case (Figure 8d) g = 50), we get a similar conclusion with scenario in (a) and (b) that the method fails to sense the flow in the center region. Overall, in all of the cases, the region near the boundary favors the measurement sensitivity hence offering the privilege over conventional particle based measurement method. We do believe the no slip boundary condition and the homeotropic anchoring condition near the boundary contribute to the large direct field gradient and hence the relatively high sensitivity.

## 5. Conclusions

In this paper, we have numerically studied director field and velocity field of two-dimensional Poiseuille flow and Couette flow. The director profile was calculated for various pressure gradient and shear stress. By solving simplified nondimensional L-E equations we obtained numerical results of pressure-driven flow and shear-driven flow.

Firstly, for pressure-driven flow, we find that (i) Limited by weak flow effects, flow alignment does not occur and the director orientation is influenced predominantly by the boundary conditions. In such cases, the velocity profile is like a parabola and symmetric about *z* = 0. (ii) When the pressure gradient arrives at a value, a qualitative change in the director profile is given explicitly.

Flow alignment occurs and orientational boundary layers exist near the planes. For shear-driven flow, (i) the results show that if the velocity is less or equal to the threshold, the director field is similar to a parabola and the velocity field is presented in a linear manner and the mid-plane angles $\emptyset_{m}$ tend to $\emptyset_{c}$ = arctan[(α_3_/α_2_)^0.5^] with the increasing velocity. (ii) When the velocity exceeds the threshold, the profiles will lose its stability. Vthreshold is about 10^−3^–10^−4^ m/s (depend on different parameters). If the velocity exceeds the threshold, the director deviates from the plane of shear. The deformation takes a form of director rotation about the axis perpendicular to the layer plane. As a result, transverse flow arises. The method of nondimensionalizing and the numerical approach proposed in this article will be a potential technique in liquid crystal research. The coupling influence what we analyze is might be able to give clues to the flow dynamics.

Flow measurement by means of the LC reorientation has been proposed recently and in this article, we have theoretically analyzed the viability of such method in flow sensing. The results show that the proposed method has great advantages for sensing near the boundaries which could complement to the technique currently adopted. In this article, we also showed that the orientation angle of the LC follows a non-linear relationship to the flow field distribution. The mathematical model proposed here could be of great potentials when the quantitatively measurement strategy is to be developed for practical applications.

## Data Availability

The data published is available upon reasonable request with the corresponding authors.

## Appendix A

It will be assumed that the director and the velocity take the forms

(A1) $$\vec{n}=[\cos\theta (z),0,\sin\theta (z)]$$

(A2) $$\vec{v}=[v(z),0,0]$$

Clearly, the constraints (1) are satisfied and because of the dependence of *z* on *n* and v, the governing LE dynamic equations in the absence of any forces F and G become

(A3) $$0={\tilde{g}}_{j}n_{j,i}-{\left(\tilde{p}+W_{F}\right)}_{i}+{\tilde{t}}_{ij,j}$$

(A4) $${\left(\frac{\partial W_{F}}{\partial n_{i,2}}\right)}_{,2}-\frac{\partial W_{F}}{\partial n_{i}}+{\tilde{g}}_{i}=\lambda n_{i}$$

where *W_F_* is the usual nematic free energy density. The non-zero contribution to the rate of strain tensor A and vorticity tensor W are

(A5) $$A_{12}=A_{21}=\frac{1}{2}v^{\prime }\left(z\right),W_{12}=-W_{21}=\frac{1}{2}v^{\prime }\left(z\right)$$

where a prime denotes differentiation with respect to *z*.

(A6) $${\tilde{t}}_{ij}=\alpha_{1}n_{k}A_{kp}n_{p}n_{j}+\alpha_{2}N_{i}n_{j}+\alpha_{3}n_{i}N_{j}+\alpha_{4}A_{ij}+\alpha_{5}n_{j}A_{ik}n_{k}+\alpha_{6}n_{i}A_{jk}n_{p}$$

(A7) $${\tilde{g}}_{i}=-\gamma_{1}N_{i}-\gamma_{2}A_{ip}n_{p}$$

Equation (7) is

(A8) $$\frac{\partial }{\partial x}(P+W_{F})=\frac{\partial {\tilde{t}}_{12}}{\partial z}$$

(A9) $$\frac{\partial }{\partial z}(P+W_{F})={\tilde{g}}_{i}n_{i,y}+\frac{\partial {\tilde{t}}_{22}}{\partial z}$$

(A10) $$\frac{\partial }{\partial z}(P+W_{F})=0$$

It is clear from Equations (12) and (14), and the dependence of ${\tilde{t}}_{12}$ upon *z*, that

(A11) $$P+W_{F}=x\frac{\partial {\tilde{t}}_{12}}{\partial z}+f_{1}(z)$$

Inserting (15) into (13) shows that

(A12) $${\tilde{t}}_{12}=az+c$$

For some constants a and c because the right-hand side of (10) is a function of z only. (taking the derivative of (12) with respect to z and the derivative of (10) with respect x will also lead to the same result.) Therefore

(A13) $$P+W_{F}=ax+f_{1}(z)$$

It is now seen from (10) and (14) that

(A14) $$f_{1}\left(z\right)=p_{0}+t_{22}+\int g_{i}n_{i,j}dz$$

For some constant p_0_, and hence, by (12), the pressure takes the form

(A15) $$P=-W_{F}+\frac{\partial {\tilde{t}}_{12}}{\partial z}x+P_{0}+{\tilde{t}}_{22}+\int {\tilde{g}}_{i}n_{i,z}dz$$

Using the form for ${\tilde{t}}_{12}$ given by (7), the result (13) may be formulated conveniently as

(A16) $$g\left(\theta \right)\frac{dv}{dz}=az+c$$

where

(A17) $$g(\theta )=\frac{1}{2}[2\alpha_{1}\sin^{2}\theta \cos^{2}\theta +(\alpha_{5}-\alpha_{2})\sin^{2}\theta +(\alpha_{3}+\alpha_{6})\cos^{2}\theta +\alpha_{4}]$$

*Φ* is the angle between *z* axis.

Equation (3) now has been reduced to (17), the pressure being available via (16) to allow this reduction.

Our attention is now turned to the remaining Equation (4). Using

(A18) $$W_{F}=\frac{1}{2}(K_{1}-K_{2}-K_{4}){\left(n_{i,i}\right)}^{2}+\frac{1}{2}K_{2}n_{i,j}n_{i,j}+\frac{1}{2}K_{4}n_{i,j}n_{j,i}$$

The equations are simplified to

(A19) $$K_{2}n_{1,22}+(K_{3}-K_{2}){\left(n_{2}^{2}n_{1,2}\right)}_{,2}+{\tilde{g}}_{1}=\lambda n_{1}$$

(A20) $$K_{1}n_{2,22}+(K_{3}-K_{2})[{\left(n_{2}^{2}n_{2,2}\right)}_{,2}-n_{2}n_{p,2}n_{p,2}]+{\tilde{g}}_{2}=\lambda n_{2}$$

The Lagrange multiplier λ can be eliminated from these two equations by multiplying (20) by *n_2_*, (21) by n_1_ and then subtracting the resulting equations to produce, after much tedious algebra, the single equation

(A21) $$K_{3}n_{2}n_{1,22}-K_{1}n_{1}n_{2,22}+{\tilde{g}}_{1}n_{2}-{\tilde{g}}_{2}n_{1}=0$$

Further substitutions for g_i_ and n_i_ using (8) and (1) finally reduce this equation to

(A22) $$2f\left(\theta \right)\frac{d^{2}\theta }{dy^{2}}+\frac{df}{d\theta }{\left(\frac{d\theta }{dz}\right)}^{2}-c\frac{\left[\gamma_{1}+\gamma_{2}\cos(2\theta \right)]}{g\left(\theta \right)}=0$$

where

(A23) $$f(\theta )=K_{1}\cos^{2}\theta +K_{3}\sin^{2}\theta \geq 0$$

This last inequality being valid because *K_1_* and *K_3_* are necessarily non-negative.

The flow v(z) in the z-direction may be induced in a sample of nematic liquid crystal confined between two parallel plates by the horizontal motion of the upper plate. In this case, the pressure p given by Equation (16) may be independent of z so that the constant a can be set to zero. Equations (3) and (4) then become

(A24) $$\frac{dv}{dz}=\frac{c}{g\left(\theta \right)}$$

(A25) $$2f\left(\theta \right)\frac{d^{2}\theta }{dz^{2}}+\frac{df}{d\theta }{\left(\frac{d\theta }{dz}\right)}^{2}-c\frac{\left[\gamma_{1}+\gamma_{2}\cos(2\theta \right)]}{g\left(\theta \right)}=0$$

where $\gamma_{1}=\alpha_{3}-\alpha_{2,\;}\gamma_{2}=\alpha_{6}-\alpha_{5}$, the $\alpha_{i}$ are constant viscosities for the NLC.

These two equations form the key starting point for the two shear problems in the following problems subsections.

## Appendix B

The energy equation is:

(A26) $$\rho \vec{v_{i}}=\rho F_{i}-{\left(p+W_{F}\right)}_{,i}+\tilde{g_{j}}n_{j,i}+G_{j}n_{j,i}+\tilde{t_{ij,j}}$$

And the conservation for the momentum is:

(A27) $${\left(\frac{\partial W_{F}}{\partial n_{i,j}}\right)}_{j}-\frac{\partial W_{F}}{\partial n_{i}}+\tilde{g_{i}}+G_{i}=\lambda n_{i}$$

We can further simplify the above two equation since we focus on 2D flow and we do not have the external force $F_{i}\;$and generalized volume force $G_{i}$, therefore the equation becomes

(A28) $$0=\tilde{g_{j}}n_{j,i}-{\left(\tilde{p}+W_{F}\right)}_{i}+\tilde{t_{\mathrm{ij},j}}$$

(A29) $${\left(\frac{\partial W_{F}}{\partial n_{i,2}}\right)}_{,2}-\frac{\partial W_{F}}{\partial n_{i}}+\tilde{g_{i}}=\lambda n_{i}$$

The term $\tilde{p}$ can be expressed as

(A30) $$\tilde{p}=-W_{F}+\frac{\partial \tilde{t_{12}}}{\partial z}x+P_{0}+\tilde{t_{22}}+\int \tilde{g_{i}}n_{i,z}dz$$

Thus, the energy equation can be obtained as

(A31) $$g\left(\theta \right)\frac{dv}{dz}=az+c$$

where

(A32) $$g\left(\theta \right)=\frac{1}{2}\left[2\alpha_{1}sin^{2}\theta cos^{2}\theta +\left(\alpha_{5}-\alpha_{2}\right)sin^{2}\theta +\left(\alpha_{3}+\alpha_{6}\right)cos^{2}\theta +\alpha_{4}\right]$$

The Equation (4) can be simplified using the equation

(A33) $${\left(\frac{\partial \omega_{F}}{\partial_{n_{i,j}}}\right)}_{,j}-\frac{\partial \omega_{F}}{\partial_{n_{i}}}=\left(K_{1}-K_{2}\right)n_{j,ji}+K_{2}n_{i,jj}+\left(K_{3}-K_{2}\right){\left(n_{j}n_{k}n_{i,k}\right)}_{,j}-\left(K_{3}-K_{2}\right)n_{j}n_{k,j}n_{k,i\;\;}\;\;\;$$

Hence, we have

(A34) $$\;K_{2}n_{1,22}+\left(K_{3}-K_{2}\right){\left(n_{2}{}^{2}n_{1,2}\right)}_{,2}+\tilde{g_{1}}=\lambda n_{1}$$

(A35) $$K_{1}n_{2,22}+\left(K_{3}-K_{2}\right)[{\left(n_{2}{}^{2}n_{2,2}\right)}_{,2}-n_{2}n_{p,2}n_{p,2}]+\tilde{g_{2}}=\lambda n_{2}$$

We can eliminate the Lagrange multiplier λ by subtraction of Equation (10)*n_2_ and Equation (11)*n_1_, after the simplification process we can have

(A36) $$K_{3}n_{2}n_{1,22}-K_{1}n_{1}n_{2,22}+g_{1}n_{2}-g_{2}n_{1}=0$$

By substation of Equation (13)

(A37) $$\tilde{g}=\left(\frac{1}{2}\left(\gamma_{1}-\gamma_{2}\right)v^{’}sin\theta ,-\frac{1}{2}\left(\gamma_{1}+\gamma_{2}\right)v^{’}cos\theta ,0\right)$$

We can further simplify the momentum equation

(A38) $$2f\left(\theta \right)\frac{d^{2}\theta }{dz^{2}}+\frac{df}{d\theta }{\left(\frac{d\theta }{dz}\right)}^{2}-c\frac{\left[\gamma_{1}+\gamma_{2}cos\left(2\theta \right)\right]}{g\left(\theta \right)}=0$$

where

(A39) $$f\left(\theta \right)=K_{1}cos^{2}\theta +K_{3}sin^{2}\theta \geq 0$$

*K* is the elastic constant for the liquid crystal and c is a constant.

For 2D pressure and slip boundary driven flow, the applied pressure is independent of x, therefore we can get the two conservative equations in the final form:

(A40) $$\frac{dv}{dz}=\frac{c}{g\left(\theta \right)}$$

(A41) $$2f\left(\theta \right)\frac{d^{2}\theta }{dz^{2}}+\frac{df}{d\theta }{\left(\frac{d\theta }{dz}\right)}^{2}-c\frac{\left[\gamma_{1}+\gamma_{2}cos\left(2\theta \right)\right]}{g\left(\theta \right)}=0$$

where $\gamma_{1}=\alpha_{3}-\alpha_{2\;,\;}\gamma_{2}=\alpha_{6}-\alpha_{5}$, $\alpha_{i}$ represents the viscous coefficients of liquid crystal. To

The above model adopts the following three assumptions

(i) one constant approximation applied to the Oseen–Zocher–Frank elastic free energy
  *K_11_* = *K_22_* = *K_33_*≡ *K*, where *K_11_* is the splay coefficient, K_22_ is the twist coefficient, and K_33_ is the bend coefficient
(ii) Parodi’s relationship
  $\alpha_{6}=\alpha_{5}+\alpha_{3}+\alpha_{2}$
(iii) The viscous coefficient${\;\alpha }_{1}$ = 0

Combining Equations (15)–(17) and the three above assumptions, we can have

(A42) $$\frac{dv}{dz}=\frac{c}{g\left(\Phi \right)}$$

(A43) $$2K\frac{d^{2}\theta }{dz^{2}}-c\frac{\left[\gamma_{1}+\gamma_{2}cos\left(2\theta \right)\right]}{g\left(\theta \right)}=0$$

where

(A44) $$g\left(\theta \right)=\frac{1}{2}\left[2\alpha_{1}sin^{2}\theta cos^{2}\theta +\left(\alpha_{5}-\alpha_{2}\right)sin^{2}\theta +\left(\alpha_{3}+\alpha_{6}\right)cos^{2}\theta +\alpha_{4}\right]$$

## Abbreviations

n	Director
$F_{i}$	External body force per unit mass
$G_{i}$	Generalized body force
**ρ**	Density
$W_{F}$	Elastic energy density for nematic
λ	Lagrange multiplier
p	Pressure
$t_{ij}$	Viscous stress
$g_{i}$	Vector
K	Free energy
$\alpha_{i}$	Viscosity coefficient
$\bar{v}$	Velocity of director
h	Channel half-width
Φ	Angle with z axis
c	Constan
g	Dimensionless pressure gradient
G	Pressure gradient
V	Velocity of the upper plate in Couette flow
g*	Critical dimensional pressure gradient
Vthreshold	Threshold velocity of the upper plane
